# Resectability of bilobar liver tumours after simultaneous portal and hepatic vein embolization *versus* portal vein embolization alone: meta-analysis

**DOI:** 10.1093/bjsopen/zrac141

**Published:** 2022-11-24

**Authors:** Remon Korenblik, Jasper F J A van Zon, Bram Olij, Jan Heil, Maxime J L Dewulf, Ulf P Neumann, Steven W M Olde Damink, Christoph A Binkert, Erik Schadde, Christiaan van der Leij, Ronald M van Dam, the DRAGON Trials Collaborative, L A Aldrighetti, L J van Baardewijk, L Barbier, C A Binkert, K Billingsley, B Björnsson, E Cugat Andorrà, B Arslan, I Baclija, M H A Bemelmans, C Bent, M T de Boer, R P H Bokkers, D W de Boo, D Breen, S Breitenstein, P Bruners, A Cappelli, U Carling, M Casellas i Robert, B Chan, F De Cobelli, J Choi, M Crawford, D Croagh, R M van Dam, F Deprez, O Detry, M J L Dewulf, R Díaz-Nieto, A Dili, J I Erdmann, J Codina Font, R Davis, M Delle, R Fernando, O Fisher, S M G Fouraschen, Å A Fretland, Y Fundora, A Gelabert, L Gerard, P Gobardhan, F Gómez, F Guiliante, T Grünberger, L F Grochola, D J Grünhagen, J Guitart, J Hagendoorn, J Heil, D Heise, E Herrero, G Hess, M Abu Hilal, M Hoffmann, R Iezzi, F Imani, N Inmutto, S James, F J Garcia Borobia, E Jovine, J Kalil, P Kingham, O Kollmar, J Kleeff, C van der Leij, S Lopez-Ben, A Macdonald, M Meijerink, R Korenblik, W Lapisatepun, W K G Leclercq, R Lindsay, V Lucidi, D C Madoff, G Martel, H Mehrzad, K Menon, P Metrakos, S Modi, A Moelker, N Montanari, J Sampere Moragues, J Navinés-López, U P Neumann, J Nguyen, P Peddu, J N Primrose, S W M Olde Damink, X Qu, D A Raptis, F Ratti, S Ryan, F Ridouani, I H M Borel Rinkes, C Rogan, U Ronellenfitsch, M Serenari, A Salik, C Sallemi, P Sandström, E Santos Martin, L Sarría, E Schadde, A Serrablo, U Settmacher, J Smits, M L J Smits, A Snitzbauer, Z Soonawalla, E Sparrelid, E Spuentrup, G A Stavrou, R Sutcliffe, I Tancredi, J C Tasse, U Teichgräber, V Udupa, D A Valenti, D Vass, T J Vogl, X Wang, S White, J F De Wispelaere, W A Wohlgemuth, D Yu, IJ A J Zijlstra

**Affiliations:** Department of Surgery, Maastricht University Medical Center, Maastricht, The Netherlands; GROW—Department of Surgery, School for Oncology and Reproduction, Maastricht University, Maastricht, The Netherlands; Department of Surgery, Maastricht University Medical Center, Maastricht, The Netherlands; Department of Surgery, Maastricht University Medical Center, Maastricht, The Netherlands; GROW—Department of Surgery, School for Oncology and Reproduction, Maastricht University, Maastricht, The Netherlands; Department of General, Visceral and Transplant Surgery, University Hospital RWTH Aachen, Aachen, Germany; Department of General, Visceral and Transplant Surgery, University Hospital Frankfurt, Goethe University Frankfurt, Frankfurt am Main, Germany; Department of Surgery, Maastricht University Medical Center, Maastricht, The Netherlands; Department of Surgery, Maastricht University Medical Center, Maastricht, The Netherlands; Department of General, Visceral and Transplant Surgery, University Hospital RWTH Aachen, Aachen, Germany; Department of Surgery, Maastricht University Medical Center, Maastricht, The Netherlands; Department of General, Visceral and Transplant Surgery, University Hospital RWTH Aachen, Aachen, Germany; NUTRIM—Department of Surgery, School of Nutrition and Translational Research in Metabolism, Maastricht University, Maastricht, The Netherlands; Department of Radiology, Cantonal Hospital Winterthur, Winterthur, Switzerland; Department of General, Visceral and Transplant Surgery, Klinik Hirslanden, Zurich, Switzerland; Department of General, Visceral and Transplant Surgery, Hirslanden Klink St. Anna Luzern, Luzern, Switzerland; Department of Radiology, Maastricht University Medical Center+, Maastricht, The Netherlands; Department of Surgery, Maastricht University Medical Center, Maastricht, The Netherlands; GROW—Department of Surgery, School for Oncology and Reproduction, Maastricht University, Maastricht, The Netherlands; Department of General, Visceral and Transplant Surgery, University Hospital RWTH Aachen, Aachen, Germany

## Abstract

**Background:**

Many patients with bi-lobar liver tumours are not eligible for liver resection due to an insufficient future liver remnant (FLR). To reduce the risk of posthepatectomy liver failure and the primary cause of death, regenerative procedures intent to increase the FLR before surgery. The aim of this systematic review is to provide an overview of the available literature and outcomes on the effectiveness of simultaneous portal and hepatic vein embolization (PVE/HVE) *versus* portal vein embolization (PVE) alone.

**Methods:**

A systematic literature search was conducted in PubMed, Web of Science, and Embase up to September 2022. The primary outcome was resectability and the secondary outcome was the FLR volume increase.

**Results:**

Eight studies comparing PVE/HVE with PVE and six retrospective PVE/HVE case series were included. Pooled resectability within the comparative studies was 75 per cent in the PVE group (*n* = 252) *versus* 87 per cent in the PVE/HVE group (*n* = 166, OR 1.92 (95% c.i., 1.13–3.25)) favouring PVE/HVE (*P* = 0.015). After PVE, FLR hypertrophy between 12 per cent and 48 per cent (after a median of 21–30 days) was observed, whereas growth between 36 per cent and 67 per cent was reported after PVE/HVE (after a median of 17–31 days). In the comparative studies, 90-day primary cause of death was similar between groups (2.5 per cent after PVE *versus* 2.2 per cent after PVE/HVE), but a higher 90-day primary cause of death was reported in single-arm PVE/HVE cohort studies (6.9 per cent, 12 of 175 patients).

**Conclusion:**

Based on moderate/weak evidence, PVE/HVE seems to increase resectability of bi-lobar liver tumours with a comparable safety profile. Additionally, PVE/HVE resulted in faster and more pronounced hypertrophy compared with PVE alone.

## Teaser

A systematic literature search was conducted to compare the effectiveness of simultaneously performed portal and hepatic vein embolization (PVE/HVE) and PVE alone. Eight observational comparative and six PVE/HVE case series were included. PVE/HVE results in a higher resectability rate and is more effective for future liver remnant growth.

## Introduction

Primary liver cancers and colorectal liver metastases (CRLM) are among the leading causes of cancer-related deaths^1,2^. Resection or ablation of the tumours in the affected liver segments are the only potentially curative treatment^3^. In patients undergoing liver resection for extensive bi-lobar disease, post-hepatectomy liver failure (PHLF) continues to be the primary cause of the primary cause of death^4–6^. In metastatic disease, only up to 20 per cent of patients are eligible for liver resection at the time of diagnosis, due to the extent of disease or an insufficient future liver remnant (FLR) volume^7–10^.

Generally, an FLR of approximately 30–40 per cent is considered sufficient, depending on the patient’s co-morbidities, underlying liver disease, and history of chemotherapy^11–13^. In patients with an estimated insufficient FLR at high risk of PHLF, FLR-hypertrophy-inducing procedures are a possibility to improve resectability^11^. Liver growth after portal vein embolization (PVE) was first reported in the 1980s^14^. In PVE, portal blood flow to the affected liver lobe later to be resected is occluded, which causes the de-portalized lobe to shrink, whereas growth is induced in the unaffected contralateral FLR^15^; however, 20–30 per cent of patients still do not qualify for surgery after PVE. Irresectability is mainly due to tumour progression during waiting time until sufficient hypertrophy has been achieved (typically 4–8 weeks) to allow resection or overall insufficient liver growth after PVE^16,17^.

More recently, several groups have developed techniques that aim for a faster and more pronounced liver hypertrophy, allowing a shorter timeframe between embolization and resection, and increase the number of patients eligible for surgery. Some of these hypertrophy-inducing techniques, however, are based on invasive surgical procedures such as associated liver partition and portal vein ligation for staged hepatectomy (ALPPS). In 2016, Guiu *et al*. introduced a new technique called liver venous deprivation (LVD). In LVD, the portal and hepatic vein of the diseased side of the liver are simultaneously occluded by using a combination of vascular plugs and glue to also occlude small collaterals^18^. Depending on the research group applying the method, different variants have been described for the same intervention. In the literature, bi-embolization, double vein embolization, radiological simultaneous portohepatic vein embolization (RASPE), and combined PVE and hepatic vein embolization (HVE) have been reported^19–21^. The latter consists of a combined PVE/HVE technique in which the portal vein is embolized (using glue and particles/plugs) and the hepatic vein is occluded by using vascular plugs simultaneously.

In 2019, Esposito *et al*. presented a systematic review evaluating the effectiveness of simultaneous performed PVE/HVE and its technical variants. They demonstrated that PVE/HVE is a safe and effective intervention to increase postoperative FLR volume, allowing 85 per cent of patients to undergo surgery^22^; however, studies comparing PVE/HVE with PVE alone could not be included as they were not available at that time. In the meantime, multiple studies comparing these two procedures have been published. The aim of this systematic review, therefore, is to provide an update of the effectiveness of PVE/HVE compared with PVE.

## Methods

This systematic review was written according to the PRISMA guidelines (*Appendix S1*)^23^. Randomized clinical trials (RCTs), retrospective single-arm cohort studies examining PVE/HVE, and cohort studies comparing PVE with PVE/HVE were included. Conference abstracts were excluded from this systematic review. Articles with patients aged 18 years and older diagnosed with primary or secondary liver cancer undergoing PVE or simultaneous PVE/HVE were considered eligible for inclusion. All technical variations of PVE and PVE/HVE were included, irrespective of embolization techniques and/or materials used. No other exclusion criteria with regard to patient characteristics were applied. Sequential portal and hepatic vein embolization, defined as a staged procedure with more than 48 h in between the PVE and HVE procedure, was excluded because hypertrophy of FLR induced by staged procedures is limited compared with simultaneous embolization^18,22^.

The primary endpoint of this systematic review was resectability, which was defined as a surgically successful liver resection procedure, irrespective of 90-day primary cause of death. Secondary outcomes of interests were FLR volume increase (absolute and per cent), degree of hypertrophy and/or kinetic growth rate (KGR), time interval between intervention and liver volumetry, time interval between embolization and surgery, reasons for non-resectability, and 90-day primary cause of death. From each included study, the indication for PVE/HVE, embolization technique and materials used were extracted.

A systematic literature search was performed in September 2022 in PubMed, Web of Science, and Embase with no restriction on date of publication. Only studies written in English meeting the selection criteria were reviewed. Keywords and/or Medical Subject Heading (MeSH) terms were formulated and adapted to individual search engines, equivalent free-text terms were used. Search strategies specific for each search engine and the MeSH and/or free-text terms used are in *Appendix S2*.

**Figure 1.**
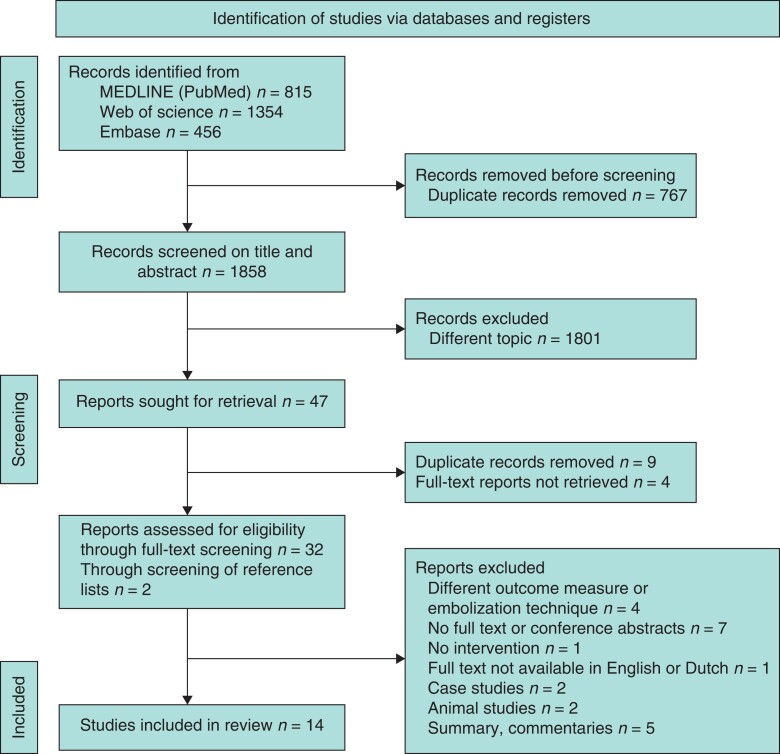
Flow chart of the applied search strategy, study selection/inclusion, and reason for exclusion

Titles and abstracts were independently screened by two reviewers (J.v.Z. and R.K.) using Rayyan software^24^. Articles were excluded if both excluded the record at the title/abstract stage. Subsequently, the same reviewers independently performed full-text screening. Disagreements regarding inclusions or exclusions of studies were resolved by discussion between the reviewers and a third reviewer (B.O.). The minimal requirement for inclusion of an article was the presentation of our primary outcome measure, resectability. Secondary outcomes that were not presented in the used articles could not be recalculated because absolute liver volumes or formula’s used were lacking. Only full-text articles on human participants in small or large cohorts were evaluated. Case reports, non-human studies, and studies in which the PVE/HVE procedure was staged (more than 48 h) were excluded. Single-arm PVE/HVE cohort studies were included only to present a comprehensive overview. The selection process is presented in a PRISMA flow chart (*Fig. 1*)^23^.

A standard data collection form designed in Microsoft^®^ Excel version 16.57 (Microsoft, Redmond, WA, USA). was used. One of the authors (J.v.Z.) extracted data from each included study. Data on the following variables were collected: sample size, patient characteristics (age, BMI, underlying liver disease status, and patient exclusion criteria), and primary and secondary outcomes. The primary outcome was expressed as a percentage.

Study quality was assessed using the Effective Public Health Practice Project (EPHPP) tool by one of the authors (J.v.Z.)^25^. Included studies were assessed on quality based on criteria in six fields: selection bias, study design, confounders, blinding, data collection methods, and withdrawal and dropouts. Quality in each field could be classified as ‘strong’, ‘moderate’, or ‘weak’. An overall rating of ‘strong’ was given by the authors according to EPHPP to studies that did not score ‘weak’ on any of the fields, ‘moderate’ to studies that had only one ‘weak’ assessment, and ‘weak’ when two or more fields were rated as ‘weak’.

Review Manager version 5.4.1 (RevMan [Computer program]. Version 5.4, The Cochrane Collaboration, 2020.) was used to pool data and calculate ORs. The Mantel–Haenszel (M–H) statistical method in combination with a fixed-effects model was used to compare the pooled resectability between groups. Results were presented in a forest plot.

## Results

A total of 1858 articles were identified by the search strategy. After screening of titles and abstracts, 47 articles were eligible for full-text screening. Four reports written in German could not be retrieved, and an additional nine duplicates were removed. Out of the resulting 34 articles, 22 were excluded after full-text screening for the following reasons: because of a sequential embolization procedure (*n* = 2)^26,27^, reporting of different outcome measures (*n* = 2), conference abstracts (*n* = 7), lack of intervention (*n* = 1), full text not available in English or Dutch (*n* = 1), case studies (*n* = 2), animal studies (*n* = 2), or summaries or commentaries (*n* = 5). Two articles were added to the review through screening of the reference lists of the articles obtained during the full-text screening^28,29^. The complete process of study identification and selection is shown in the flow chart (*Fig. 1*)^23^. Overall, this process resulted in a total of 14 studies, eight comparative studies^20,21,28–32^ and six case series^18,33–36^. The included studies were published between 2016 and 2022. Two comparative studies, PVE/HVE *versus* ALLPS^35,37^, were included in this systematic review and handled as two case series on PVE/HVE.

The characteristics of the patients are presented in *Table 1*. Sample size of the included studies ranged from 6 to 160 for PVE and 6 to 39 participants for PVE/HVE. The mean age ranged from 55 to 67 years across the intervention groups^29,32^. Three studies included patients with underlying liver disease, such as liver steatosis, fibrosis, and cirrhosis^21,31,32^. More patients with underlying liver disease were included in the PVE/HVE groups. The retrospective DRAGON Trial Collaborative analysis was the only study that reported a significant difference in underlying liver disease between the two groups (*P* = 0.021), but multivariable analysis showed that this difference did not impact the resectability rate^21^. In the other four studies, underlying liver disease status was not documented in two studies^28,38^ and was an explicit exclusion criterion in four studies^18,20,29,33^. A total of 334 patients in the PVE group and 190 patients in the PVE/HVE group were included by the comparative studies. Data from 175 PVE/HVE patients from an additional six cohort studies were included in this systematic review^18,33–36^.

**Table 1:** Study characteristics of comparative studies on portal vein embolization *versus* portal and hepatic vein embolization and single-arm case studies on portal and hepatic vein embolization

Author	Year	*n*	Tumour type(s)		Age (years)		Underlying liver disease		BMI		Exclusion criteria
			PVE	PVE/HVE	PVE	PVE/HVE	PVE	PVE/HVE	PVE	PVE/HVE	
**Comparative studies**											
Hocquelet et al.^38^	2018	PVE: 6 PVE/HVE: 6	pHCC: 6	pHCC: 6	62 (54–58)	60 (54–71)	NR	NR	NR	NR	NA
Panaro et al.^28^	2019	PVE: 16 PVE/HVE: 13	CRLM: 5 HCC: 9 Other: 2	CRLM: 10 HCC: 3	NR	NR	NR	NR	NR	NR	NA
Le Roy et al.^31^	2020	PVE: 41 PVE/HVE: 31	CRLM: 26 HCC: 2 pHCC: 2	CRLM: 10 HCC: 2 pHCC: 8	63 (60–68)	66 (55–70)	cirrhosis: 4 (10%) NASH: 11 (27%)	cirrhosis: 3 (10%) NASH: 9 (29%)	24 (23–29)	24 (23–27)	NA
Kobayashi et al.^32^	2020	PVE: 39 PVE/HVE: 21	NR	CRLM: 18 HCC: 5 iHCC: 2 pHCC: 5 Other: 1	65 (41–75)	65 (25–85)	cirrhosis: 1 (2.6%) fibrosis: 9 (23%) steatosis:14(36%)	cirrhosis: 0 (0%) fibrosis: 8 (38%) steatosis: 8 (38%)	23.8 (17.1–33.5)	23.4 (18.9–36.3)	NA
Laurent et al.^20^	2020	PVE: 36 PVE/HVE: 37	CRLM: 20 iHCC: 7 HCC: 4 NET: 3	CRLM: 23 iHCC: 7 HCC: 4 NET: 2	60.92 (51–72)	64.41 (61–71)	NR	NR	25.54(6)	25.41(7)	Liver fibrosis and cirrhosis
Guiu et al.^29^	2020	PVE: 22 PVE/HVE: 29	CRLM: 17 iHCC: 3 HCC: 1 Other: 1	CRLM: 22 iHCC: 4 HCC: 2 Other: 1	66 (45–79)	62 (26–79)	NR	NR	25.1 (16–35.2)	26.3 (17.6–34.5)	Klatskin tumour, liver cirrhosis
Heil et al.^21^	2021	PVE: 160 PVE/HVE: 39	CRLM: 85 HCC: 11 iHCC: 22 pHCC: 25 GBC: 9 Other: 8	CRLM: 19 HCC: 4 iHCC: 4 pHCC: 5 GBC: 4 Other: 3	67 (58–73)	63 (52–67)	cirrhosis: 13 (8.1%) fibrosis: 22 (14%) steatosis: 37 (23%)	cirrhosis: 1 (2.6%) fibrosis: 1 (2.6%) steatosis:17(44%)	25.2 (23–28.3)	24.4 (22.7–26.9)	NA
Boning et al.^39^	2022	PVE: 14 PVE/HVE: 14	CRLM: 4 pHCC: 10	CRLM: 4 pHCC: 10	65.1(11.4)	68.1(10.5)	NR	NR	26.1(4.2)	24.1(3.6)	NA
**Case series**											
Guiu et al.^18^	2016	PVE/HVE: 7	NA	CRLM: 2 HCC: 1 iHCC: 3 pHCC: 1	NA	63.6 (42–77)	NA	NR	NA	NR	Liver cirrhosis
Guiu et al.^33^	2017	PVE/HVE: 10	NA	CRLM: 7 pHCC: 1 Other: 2	NA	60.5 (46–71)	NA	NR	NA	NR	Liver cirrhosis
Le Roy et al.^19^	2017	PVE/HVE: 7	NA	CRLM: 2 iHCC: 1 pHCC: 2 Other: 2	NA	62.6	NA	NR	NA	NR	NA
Chebaro et al.^35^	2021	PVE/HVE: 124	NA	CRLM: 15 pHCC: 16 Other: 3	NA	66 (39–83)	NA	NR	NA	32(5.4)	NA
Ghosn et al.^40^	2021	PVE/HVE: 12	NA	CRLM: 12	NA	55.5(11.8)	NA	NR	NA	NR	NA
Cassese et al.^37^	2022	PVE/HVE: 15	NA	CRLM: 14 Other: 1	NA	58.7	NA	NR	NA	25.9(3.4)	Liver cirrhosis

All values are median (range or i.q.r.) or mean(s.d.) as extracted from the included studies. PVE, portal vein embolization; PVE/HVE, simultaneous PVE and hepatic vein embolization; CRLM, colorectal cancer liver metastasis; HCC, hepatocellular carcinoma; iHCC, intrahepatic carcinoma; pHCC, perihilar carcinoma; GBC, gallbladder carcinoma; NET, neuroendocrine tumour; NR, not reported; NA, not applicable; NASH, non-alcoholic steatohepatitis; i.q.r., interquartile range.

Information on selection of baseline per cent FLR, definition of PHLF, additional segment four embolization, additional middle hepatic vein occlusion, and formulae used for baseline FLR are presented in *Table S1*.

**Figure 2.**
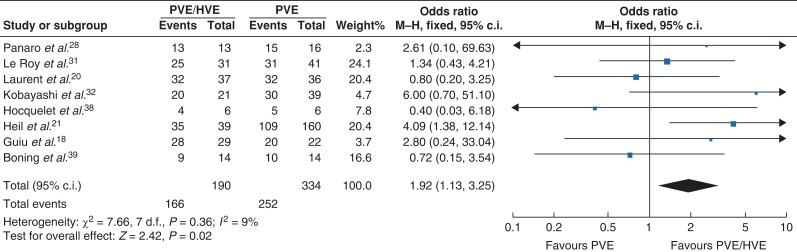
Forest plot of calculated ORs (95% c.i.) for resectability in comparative studies PVE, portal vein embolization; PVE/HVE, simultaneous PVE and hepatic vein embolization.

Resectability rates are presented in *Table 2*. In most studies, resectability did not differ significantly between treatments and ranged from 64 to 94 per cent and from 67 to 100 per cent for PVE and PVE/HVE respectively. Summary analysis showed that resection was possible in 252 (75 per cent) patients after PVE and 166 (87 per cent) patients after PVE/HVE in the comparative studies. In the single-arm studies, 175 patients underwent PVE/HVE, of whom 146 patients underwent successful liver resection (83 per cent). Overall, reasons for non-resectability were insufficient post-embolization FLR volume in 22 (6.6 per cent) *versus* 5 (1.4 per cent) patients, peritoneal carcinomatosis in 11 (0.3 per cent) *versus* 5 (1.4 per cent) patients, or disease progression in 47 (14.1 per cent) *versus* 38 (10.4 per cent) patients, for PVE and PVE/HVE respectively. In the retrospective DRAGON analysis, a significant difference in resectability was found between groups (*P* = 0.007)^21^. Kobayashi *et al*.^32^ did not present a *P* value for resectability, so it is uncertain whether the difference was statistically significant in this article. Other studies reported non-significant differences or did not report significance. For each comparative study, ORs (with 95 per cent confidence intervals) for resectability were calculated and are graphically presented in *Fig. 2*. After pooling of results, resectability was more frequently possible after PVE/HVE (OR 1.92 (1.13 to 3.25), *P* = 0.015) with a 9 per cent heterogeneity (*I*^2^) across the included studies (*Fig. 2*). Eleven out of 14 articles reported on 90-day primary cause of death^21,29–37,39^. In the comparative studies, 90-day primary cause of death was similar between the groups (2.53 per cent after PVE *versus* 2.23 per cent after PVE/HVE respectively), whereas a higher 90-day primary cause of death was reported in the single-arm PVE/HVE cohort studies (6.9 per cent, 12 of 175 patients).

**Table 2:** Waiting time, resectability rates, and 90-day primary cause of death

Author	Time between embolization and surgery (days)		Resectability (%)		Reasons for exclusion from resection		90-day primary cause of death	
	PVE	PVE/HVE	PVE	PVE/HVE	PVE	PVE/HVE	PVE	PVE/HVE
**Comparative studies**								
Hocquelet et al.^38^	NR	21	83	67	peritoneal carcinomatosis: 1	extrahepatic disease progression: 2	2	0
Panaro et al.^28^	37	38	94	100	peritoneal carcinomatosis: 1	NA	NR	NR
Le Roy et al.^31^	NR	NR	76	81	insufficient FLR-V: 2 disease progression: 5	disease progression: 3 peritoneal carcinomatosis: 3	2	3
Kobayashi et al.^32^	35 (20–181)	35 (23–109)	77	95	disease progression: 9	disease progression: 1	0*	0*
Laurent et al.^20^	44 (21–78)	36 (16–47)	89	86	insufficient FLR-V: 1 disease progression: 3	intrahepatic disease progression: 5	NR	NR
Guiu et al.^29^	36 (22–55)	32 (22–46)	91	97	insufficient FLR-F: 2	insufficient FLR-F: 1	1	0
Heil et al.^21^	41 (28–61)	37 (21–52)	68	90	insufficient FLR-V: 17 (5 underwent rescue HVE) disease progression: 31 postinterventional complications: 3	insufficient FLR-V: 1 disease progression: 2 postinterventional complications: 1	3	1
Boning et al.^39^	30.57(6.86)	30.50(7.17)	71	64	Tumour progression: 4	Tumour progression: 5	5	3
**Case series**								
Guiu et al.^18^	NA	23 (13–30)	NA	85.6	NA	peritoneal carcinomatosis: 1	NA	NR
Guiu et al.^33^	NA	31 (22–45)	NA	90	NA	NR	NA	0
Le Roy et al.^19^	NA	49 (20–210)	NA	85.6	NA	peritoneal carcinomatosis: 1	NA	1
Chebaro et al.^35^	NA	37 (15–1015)	NA	80.6	NA	disease progression: 20 perioperative deaths: 14	NA	10
Ghosn et al.^40^	NA	39(7.5)	NA	83.3	NA	disease progression: 1 insufficient %FLR: 1	NA	1
Cassese et al.^37^	NA	39 (23–57)	NA	100	NA	NA	NA	0

Values are median (range or i.q.r.), or mean(s.d.) as extracted from the included studies. PVE, portal vein embolization; PVE/HVE, simultaneous portal and hepatic vein embolization; FLR-V, future remnant liver volume; FLR-F, future remnant function; NR, not reported; NA, not applicable. *Not clear whether this is the 30-day or 90-day primary cause of death.

Median days between embolization and resection ranged from 35–44 days for PVE and 23–49 days for PVE/HVE with considerable variation.

Liver volume before and after embolization, time interval between embolization and liver volumetry, and mean or median KGR are presented in *Table 3*. At baseline pre-embolization, FLR volume was expressed in ml or cc, and either per cent FLR^20,29,32,38^ or per cent standardized FLR (sFLR)^21,32^. A comparison of the studies on FLR volume was difficult due to the usage of different volume metrics. All studies reported an increase in FLR volume and percentage from baseline after both PVE and PVE/HVE. Baseline FLR volume ranged from 294 to 542 ml for PVE and from 281 to 547 ml for PVE/HVE. A significant difference was reported for per cent FLR at baseline between the groups only in the study by Laurent *et al*. (*P* < 0.001)^20^.

**Table 3:** Volumetry and growth data

Author	Baseline FLR volume (ml, cc, %)		Time between embolization and liver volumetry (days)		Hypertrophy (%)		KGR	
	PVE	PVE/HVE	PVE	PVE/HVE	PVE	PVE/HVE	PVE	PVE/HVE
**Comparative studies**								
Hocquelet et al.^38^	429 (391–560) 31 FLR (24–33)	517 (310–828) 30.5 FLR (23–35.5)	23.5* (15–29)	23.5* (15–29)	31.3 (10.57–43.27)	67 (13–148)	NR	NR
Panaro et al.^28^	NR	31.2(6.5) FLR	21*	21*	NR	40.8(7.9)% FLR†	4.8(4) cc/day	16(7) cc/day
Le Roy et al.^31^	348 (266–547)	394 (262–478)	27	26	31.9(34)	51.2(41.7)	8(13)%/week	19(18)%/week
Kobayashi et al.^32^	523 (420–659) 24 sFLR (20–30	547 (453–656) 25 sFLR (32–31)	26 (17–33)	22 (17–30)	12 (5–23)	36 (26–53)	1.4%/week (0.7–2.1)	2.9%/week (1.9–4.3)
Laurent et al.^20^	468 (253–945) 31.03 FLR (18.33–38.95)	387 (200–623) 22.91 FLR (16.55–32.15)	30 (25–43)	31 (21–40)	29	61.2	NR	NR
Guiu et al.^29^	542 (236–1119) 27.4 FLR (13.7–47.7)	484 (233–805) 22.6 FLR (16.6–37.7)	21	21	18.6 (−10.7–102.2)	52.6 (1.0–175.6)	NR	NR
Heil et al.^21^	294 (233–389.7) 18.5 sFLR (15–25)	281 (234–352.1) 18 sFLR (16–23)	24 (19–37)	17 (13–32)	48 (24–69)	59 (45–79)	2.5%/week (1.1–3.8)	3.5%/week (2.2–7.1)
Boning et al.^39^	NR	NR	30*	30*	44.9(28.9)	48.2(22.2)	NR	NR
**Case series**								
Guiu et al.^18^	NA	409 (345–601) 28.2% FLR (22.4–33.3)	NA	23 (13–30)	NA	NR	NA	4.2%/week
Guiu et al.^33^	NA	332(59) 20.8(5.1) sFLR	NA	21	NA	63.3	NA	25 cc/day(8)
Le Roy et al.^19^	NA	389 (182–508) 21 FLR (14–37)	NA	22 (19–28)	NA	52.6 (18.2–187.9)	NA	18%/week
Chebaro et al.^35^	NA	379 (161–961)	NA	28 (4–52)	NA	63	NA	2%/day
Ghosn et al.^40^	NA	505(125) 28.7(5.9) FLR	NA	28(7.6)	NA	48.5(24)	NA	3.56%/week(2.3)
Cassese et al.^37^	NA	29.3(6.8)	NA	21	NA	49(29)	NA	0.2%/day(0.2)

Values are median (range or i.q.r.), or mean(s.d.) as extracted from the included studies. FLR, future liver remnant; KGR, kinetic growth rate; PVE, portal vein embolization; PVE/HVE, simultaneous portal and hepatic vein embolization; NR, not reported. *Authors did not provide specific numbers for each group, only a mean was presented. †No information was given on FLR hypertrophy, instead post-embolization FLR percentage was presented.

Contrary to baseline FLR, post-embolization FLR volume was expressed in ml or cc by five studies, whereas two studies only reported on the degree of hypertrophy^28,29^. FLR volume after embolization ranged between 442 and 696 ml for PVE, and between 470 and 845 ml for PVE/HVE. Per cent FLR ranged from 39 to 43 per cent after PVE and 39 to 43 per cent after PVE/HVE. Per cent sFLR ranged from 28 to 31 per cent after PVE and 31 to 36 per cent after PVE/HVE.

Different formulae were used to calculate liver hypertrophy; (FLR_after__−_ FLR_before_)/FLR_before_^30^, (FLR_after_: total liver volume_after_)/(FLR_before_: total liver volume_before_)^32^, or volume FLR_before_: FLR_after_^21^. Where degree of hypertrophy was defined as either sFLR_after__−_ sFLR_before_^21,32^, volume FLR_after_: volume FLR_before_^20,31^, or per cent FLR_after −_per cent FLR_before_^40^. After PVE, a growth between 12 and 48 per cent (after a median of 21–30 days) was observed, whereas growth between 36 and 67 per cent was reported after PVE/HVE (after a median of 17–31 days), with four studies finding significant differences between the two groups^21,29,31,32^. Two studies did not report on per cent hypertrophy^18,28^.

Mean or median time (days) between embolization and liver volumetry was 21–30 and 17–31 days for PVE and PVE/HVE respectively. Heil *et al*. (2021) reported a significant difference on interval between embolization and liver volumetry, favouring the PVE/HVE group (24 *versus* 17 days, *P* = 0.009). Laurent *et al*. (2020) showed a non-significant difference (*P* = 0.95)^20^, whereas the remaining studies did not report on significance.

Three studies reported a significant difference on KGR between PVE and PVE/HVE (all *P* < 0.001) ^21,29,32^. Le Roy *et al*. (2020) presented liver growth as degree of hypertrophy: 8 per cent *versus* 19 per cent, for PVE and PVE/HVE respectively (*P* = 0.026)^31^. These rates were comparable to the degree of hypertrophy of 13 per cent *versus* 21 per cent for PVE and PVE/HVE reported by Heil *et al*. (*P* < 0.001)^21^.

An overview of the study quality assessment of the included studies is presented in *Table 4*. According to EPHPP, two articles were rated as ‘weak’^28,38^, and the others as ‘moderate’. No RCTs were included in this systematic review and all the studies reported on small numbers.

**Table 4:** The Effective Public Health Practice Project quality assessment for included studies

Author	Selection bias	Study design	Confounders	Blinding	Data collection methods	Withdrawals and dropouts	Global rating
Hocquelet et al.^38^	-	±	-	±	±	-	Weak
Panaro et al.^28^	-	±	-	±	+	+	Weak
Le Roy et al.^31^	-	±	+	±	+	+	Moderate
Kobayashi et al.^32^	-	±	+	±	+	±	Moderate
Laurent et al.^20^	-	±	+	±	+	±	Moderate
Guiu et al.^29^	-	±	+	±	+	+	Moderate
Heil et al.^21^	-	±	+	±	+	+	Moderate
Boning et al.^39^	-	±	-	±	+	+	Weak
Guiu et al.^18^	-	±	-	±	+	+	Weak
Guiu et al.^33^	-	±	-	±	+	+	Weak
Le Roy et al.^19^	-	±	-	±	+	-	Weak
Chebaro et al.^35^	-	±	-	±	-	-	Weak
Ghosn et al.^40^	-	±	-	±	+	+	Weak
Cassese et al.^37^	-	±	-	±	+	+	Weak

Weak quality score (-) , Moderate quality score(±), strong quality score (+).

## Discussion

This systematic review and meta-analysis shows that PVE/HVE, as a regenerative procedure to enhance FLR hypertrophy in patients scheduled for extended liver resection, results in a significantly higher resection rate (87 *versus* 75 per cent) compared with PVE only. The KGR tends to be higher in the PVE/HVE group but could not be statistically analysed due to the different ways of reporting this variable by different studies. The 90-day primary cause of death was comparable in the two embolization groups. These results suggest superiority of PVE/HVE over PVE in regenerative effectiveness; however, this systematic review included only retrospective studies of moderate or weak quality at best. In addition, only a small number of participants was included in most of the studies.

The relatively large multicentre study of Heil *et al*. was the only study able to demonstrate a significant difference in liver resectability, which was primarily responsible for the results of the pooled primary outcome of this meta-analysis^21^. The lack of difference in resectability presented by most of the studies individually may be explained by relatively large FLR volumes before the embolization, long intervals between embolization and resection, small sample sizes, patient selection, use of interval chemotherapy, or differing practice regarding chemotherapy-free intervals. Patients with CRLM who are kept off chemotherapy for long intervals before complete resection may have a propensity of progression in the process of waiting.

In another review by Heil *et al*. a resection success rate of 87 per cent after PVE/HVE was reported^41^. An 85 per cent resection rate was reported after PVE/HVE by Esposito *et al*.^22^. Both their reviews included studies with patients with multiple tumour types. In the present systematic review, resection rates after PVE/HVE ranged from 67 to 100 per cent. This demonstrates that the rates vary widely depending on the type of study and the number of patients who were included and are influenced by selection and treatment biases in each centre.

We recommend researchers to use one standardized growth outcome measure in future publication to be able to compare future studies more easily. We propose to use KGR as this is a standardized outcome measure. KGR reflects the regenerative capacity of the FLR over time and therefore can be compared more reliably among different studies. The KGR also has a predictive value for the potential risk for future PHLF^42^; however, KGR as an outcome measure carries the risk of bias as the majority of growth is achieved in the first week after embolization, until a plateau phase is reached after approximately 21 days, as shown by two studies in the present meta-analysis^17,31,38^. A consensus among included studies seems to exist to obtain volumetric data 21 days or less after embolization. A longer waiting interval until volumetry consequently leads to underestimation of the KGR when compared with a KGR with a shorter time interval. Therefore, standardization of time intervals after both PVE and PVE/HVE is required for adequate comparison. We recommend researchers to present the KGR over the first 3 weeks after embolization in future publications. Furthermore, the difference in baseline FLR percentage should be accounted for, as studies that included patients with lower baseline FLR percentage displayed a higher KGR in the first weeks compared with studies that included patients with higher baseline FLR percentages.

The potential that surgery may be performed earlier after PVE/HVE may be a further advantage of this combined procedure, which may translate into long-term oncological survival benefits. Although, the included studies probably did not focus on decreasing the time from intervention to surgery, as shown by the long interval, the interval was reported to be significantly shorter by Heil *et al*.^21^.

The time interval to obtain volumetric data in the included studies suggests that performing the first postembolization volumetry at week 1 can shorten the interval time between embolization and surgery. In cases where the FLR is not sufficient at 1 week after embolization volumetry, an estimation of the eventually expected FLR hypertrophy can be made based on the KGR. Consequently, the smallest time window to resection can be achieved to further reduce the risk of tumour progression and increase the feasibility of resection.

There are some limitations regarding the included studies and the systematic review itself. First, in contrast to a previous systematic review^22^ and a scoping review^41^ it was decided to present outcome measures in several metrics, so that the included studies could be compared more easily. This showed that research groups opt for various formulae to calculate outcome measures. In particular, the formula to calculate the outcome measure ‘hypertrophy’ was not consistent across the included studies, therefore readers of the present systematic review should be cautious about the interpretation of this outcome. This is a major concern and hinders an accurate comparison between reported results. For reliable comparison of future studies, the formula sFLR1 − sFLR0 for the degree of hypertrophy is recommended by the authors of this systematic review as well as the formula for KGR: $\frac{\mathrm{sFLR}1-\mathrm{sFLR}0}{\mathrm{Interval}\;\mathrm{between}\;\mathrm{embolization}\;\mathrm{and}\;\mathrm{resection}\;\mathrm{in}\;\mathrm{weeks}}$.

Another limitation of this review is the small sample size in the different studies. Third, included articles were best qualified as ‘moderate’ according to the EPHPP. The risk of selection bias therefore is very high due to the retrospective design, and it is often difficult or even impossible to determine how the authors dealt with this issue.

Although patients are selected for embolization based on guidelines obtained by CT volumetry^11–13^, several studies included patients in the PVE group with high baseline FLR percentages^20,32,38^. This suggests that patients with higher baseline FLRs (25–30 per cent) are ideal candidates, but further research needs to clarify cutoffs in different types of liver quality and/or function. Laurent *et al.* reported that one-third of the included patients had no initial indication for embolization to induce liver hypertrophy before liver resection^20^. This raises questions about the selection of patients as appropriate controls in these studies. Therefore, it is recommended that both KGR and baseline FLR volumes are reported. Moreover, choosing an appropriate matched control group seems to be important to allow for valid comparison, as two studies had relatively large differences between the control and intervention group^20,29^. Furthermore, an era bias was observed in some studies, as PVE was completely replaced by PVE/HVE^32,38^. This observation is supported by the fact that the same selection criteria were used for PVE and PVE/HVE. Considering the assumption that PVE/HVE induces faster and more growth, some might have expected lower baseline per cent FLR in the PVE/HVE group; however, baseline per cent FLR did not statistically differ in most of the included studies.

RCTs with larger sample sizes are needed to make reliable statements on the actual effect of PVE/HVE compared with PVE. Two prospective trials are currently in progress, the HYPER-LIV01 (registration number: NCT03841305; http://www.clinicaltrials.gov) and DRAGON 1 trial (registration number: NCT04272931; http://www.clinicaltrials.gov)^43,44^. The global multicentric DRAGON 2 RCT (registration number: NCT05428735; (http://www.clinicaltrials.gov) was just ethically approved and will commence later this year.

PVE/HVE seems to be more effective than PVE with regard to resection rate and seems to offer increased KGR. Therefore, we suggest that, to induce hypertrophy before major liver resection, PVE/HVE could be considered in patients with a small FLR. Prospective trials to examine the exact role of PVE/HVE are currently underway.

## Collaborators

**The DRAGON Trials Collaborative**

L. A. Aldrighetti (IRCCS San Raffaele Hospital, Milano, Italy); L. J. van Baardewijk (Maxima Medical Center, Eindhoven, The Netherlands); L. Barbier (Auckland District Health Board, Auckland, New Zealand); C. A. Binkert (Kantonsspital (KSW) Winterthur, Winterthur, Switzerland); K. Billingsley (Yale school of Medicine, New Haven, USA); B. Björnsson (University hospital of Linköping, Linköping, Sweden); E. Cugat Andorrà (Hospital Universitari Germans Trias i Pujol de Badalona, Barcelona, Spain); B. Arslan (Rush University Medical Center, Chicago, USA); I. Baclija (Kaiser Franz Josef Hospital, Vienna, Austria); M. H. A. Bemelmans (Maastricht University Medical Center+, Maastricht, The Netherlands); C. Bent (Royal Bournemouth and Christchurch Hospital, Bournemouth, UK); M. T. de Boer (University Medial Center Groningen, Groningen, The Netherlands); R. P. H. Bokkers (University Medical Center Groningen, Groningen, The Netherlands); D. W. de Boo (Monash Health, Clayton, Australia); D. Breen (University Hospital Southampton, Southampton, UK); S. Breitenstein (Kantonsspital (KSW) Winterthur, Winterthur, Switzerland); P. Bruners (University Hospital Aachen, Aachen, Germany); A. Cappelli (Sant’Orsola-Malpighi Hospital, University of Bologna, Bologna, Italy); U. Carling (Oslo University Hospital, Oslo, Norway); M. Casellas i Robert (Dr. Josep Trueta, Girona, Spain); B. Chan, (Univeristy Hospital Aintree, Liverpool, UK); F. De Cobelli, (IRCCS Ospedale San Raffale, Milano, Italy); J. Choi (Western Health, Melbourne, Australia); M. Crawford (Royal Prince Alfred Hospital, Sydney, Australia); D. Croagh (Monash Health, Clayton, Australia); R. M. van Dam (Maastricht University Medical Center+, Maastricht, The Netherlands); F. Deprez (CHU- UcLouvain-Namur, Yvoir, Belgium); O. Detry (CHU Liege, University of Liege, Belgium); M. J. L. Dewulf (Maastricht University Medical Center+, Maastricht, The Netherlands); R. Díaz-Nieto (Aintree University Hospital, Liverpool, UK); A. Dili (CHU-UCL Namur site Godine, Namur, Belgium); J. I. Erdmann (Amsterdam University Medical Centers, Amsterdan, The Netherlands); J. Codina Font (University Hospital Dr. Josep Trueta Girona, Spain); R. Davis (Aintree University Hospital, Liverpool, UK); M. Delle (Karolinska University Hospital, Stockholm, Sweden); R. Fernando (Auckland District Health Board, Auckland, New Zealand); O. Fisher (Royal Prince Alfred Hospital, Sydney, Australia); S. M. G. Fouraschen (University Medial Center Groningen, Groningen, The Netherlands); Å. A. Fretland (Oslo University Hospital, Oslo, Norway); Y. Fundora (Clínic Barcelona, Barcelona, Spain); A. Gelabert (Parc Tauli Hospital, Sabadell, Spain); L. Gerard (CHU Liege, Belgium); P. Gobardhan (Amphia, Breda, The Netherlands); F. Gómez (Clínic de Barcelona, Barcelona, Spain); F. Guiliante (Catholic University Rome, Rome, Italy); T. Grünberger (Kaiser Franz Josef Hospital, Vienna, Austria); L. F. Grochola (Kantonsspital (KSW) Winterthur, Winterthur, Switzerland); D. J. Grünhagen (Erasmus Medical Center, Rotterdam, The Netherlands); J. Guitart (University Hospital Mútua Terassa, Terassa, Spain); J. Hagendoorn (University Medical Center Utrecht, Utrecht, The Netherlands); J. Heil (Goethe-University Frankfurt, University Hospital Frankfurt, Germany); D. Heise (University Hospital Aachen, Aachen, Germany); E. Herrero (University Hospital Mútua Terassa, Terassa, Spain); G. Hess (Clarunis, University Centre for Gastrointestinal and Liver Diseases, Basel, Switzerland); M. Abu Hilal (Fondazione Poliambulanza Hospital Institute, Brescia, Italy); M. Hoffmann (St. Clara hospital, Basel, Switzerland); R. Iezzi (Agostino Gemelli University Hospital Rome, Rome, Italy); F. Imani (Amphia, Breda, The Netherlands); N. Inmutto (Maharaj Nakorn Chiang Mai Hospital, Chiang Mai, Thailand); S. James (Maastricht University Medical Center+, Maastricht, The Netherlands); F. J. Garcia Borobia (Parc Tauli Hospital, Sabadell, Spain); E. Jovine (Maggiore Hospital, Bologna, Italy); J. Kalil (McGill University Health Centre, Montreal, Canada); P. Kingham (Memorial Sloan Kettering Cancer Center, New York, USA); O. Kollmar (Clarunis University Centre for Gastrointestinal and Liver Diseases, Basel, Switzerland); J. Kleeff (University Hospital Halle (Saale), Halle (Saale), Germany); C. van der Leij (Maastricht University Medical Center+, Maastricht, The Netherlands); S. Lopez-Ben (University Hospital Dr. Josep Trueta, Girona, Spain); A. Macdonald (Oxford University Hospitals, Oxford, UK); M. Meijerink ( Amsterdam University Medical Centers, Amsterdam, The Netherlands); R. Korenblik (Maastricht University Medical Center+, Maastricht, The Netherlands); W. Lapisatepun (Maharaj Nakorn Chiang Mai Hospital, Chiang Mai, Thailand); W. K. G. Leclercq (Maxima Medical Center, Eindhoven, The Netherlands); R. Lindsay (Belfast Health and Social Care Trust, Belfast, UK); V. Lucidi (Erasme University Hospital – ULB, Brussels, Belgium); D. C. Madoff (Yale school of Medicine, New Haven, USA); G. Martel (Ottawa hospital, Ottawa, Canada); H. Mehrzad (Queen Elisabeth Hospital Birmingham, University Hospital Birmingham, Birmingham, UK); K. Menon (King’s College Hospital, London, UK); P. Metrakos (McGill University Health Centre, Montreal, Canada); S. Modi (University Hospital Southampton, Southampton, UK); A. Moelker ( Erasmus MC University Medical Center, Rotterdam, The Netherlands); N. Montanari, (Maggiore Hospital, Bologna, Italy); J. Sampere Moragues (Hospital Universitari Germans Trias i Pujol de Badalona, Barcelona, Spain); J. Navinés-López (Hospital Universitari Germans Trias i Pujol de Badalona, Barcelona); U. P. Neumann (University Hospital Aachen, Aachen, Germany); J. Nguyen (Western Health, Melbourne, Australia); P. Peddu (King’s College Hospital, London, UK); J. N. Primrose (University Hospital Southampton, Southampton, UK); S. W. M. Olde Damink (Maastricht University Medical Center+, Maastricht, The Netherlands); X. Qu (Zhongshan hospital, Fundan University, Shanghai, China); D. A. Raptis (Royal Free Hospital, London, UK); F. Ratti (IRCCS Ospedale San Raffaele, Milan, Italy); S. Ryan (Ottawa hospital, Ottawa, Canada); F. Ridouani (Memorial Sloan Kettering Cancer Center, New York, USA); I. H. M. Borel Rinkes (University Medical Center Utrecht, Utrecht, The Netherlands); C. Rogan (Royal Prince Alfred Hospital, Sydney, Australia); U. Ronellenfitsch (University Hospital Halle (Saale), Halle (Saale), Germany); M. Serenari (Sant’Orsola-Malpighi Hospital, University of Bologna, Bologna, Italy); A. Salik (University hospital of Linköping, Linköping, Sweden); C. Sallemi (poliambulanza, Brescia, Italy); P. Sandström (University hospital of Linköping, Linköping, Sweden); E. Santos Martin (Memorial Sloan Kettering Cancer Center, New York, USA); L. Sarría, (Miguel Servet University Hospital and University of Zaragoza, Zaragoza, Spain); E. Schadde (Klinik Hirslanden, Luzern and Zurich, Switzerland); A. Serrablo (Miguel Servet University Hospital Zaragoza, Zaragoza, Spain); U. Settmacher (University Hospital Jena, Jena, Germany); J. Smits (Maastricht University Medical Center+, Maastricht); M. L. J. Smits (University Medical Center Utrecht, Utrecht, The Netherlands); A. Snitzbauer (Goethe-University Frankfurt, University Hospital Frankfurt, Germany); Z. Soonawalla (Oxford University Hospitals, Oxford, UK); E. Sparrelid (Karolinska Institutet, Karolinska University Hospital, Stockholm, Sweden); E. Spuentrup (Hospital Saarbrücken, Saarbrücken, Germany); G. A. Stavrou (Hospital Saarbrücken, Saarbrücken, Germany); R. Sutcliffe (University Hospital Birmingham, Birmingham, UK); I. Tancredi (Erasme University Hospital – ULB, Brussels, Belgium); J. C. Tasse (University Medical Center, Chicago, USA); U. Teichgräber (University Hospital Jena, Jena, Germany); V. Udupa (Oxford University Hospitals NHS Foundation trust, Oxford, UK); D. A. Valenti (McGill University Health Centre, Montreal, Canada); D. Vass (Belfast Health and Social Care Trust, Belfast, UK); T. J. Vogl (Goethe-University Frankfurt, University Hospital Frankfurt, Germany); X. Wang (Zhongshan hospital, Fudan University, Shanghai, China); S. White (The Newcastle upon Tyne Hospitals, Newcastle, UK); J. F. De Wispelaere (CHU-UCL Namur site Godinne, Namur, Belgium); W. A. Wohlgemuth (University of Halle-Wittenberg, Halle (Saale), Germany); D. Yu (Royal Free Hospital London, London, UK); IJ. A. J. Zijlstra (Amsterdam University Medical Centers, Amsterdam, The Netherlands).

## Supplementary Material

zrac141_Supplementary_Data

## Data Availability

All data generated or analysed during this study are included in this published article and its supplementary information files.
