# Supplementary material for: Resectability of bilobar liver tumours after simultaneous portal and hepatic vein embolization versus portal vein embolization alone: meta-analysis
Source: BJS Open. 2022 Nov 24;6(6):zrac141. doi: 10.1093/bjsopen/zrac141 (PMC9702575; doi:10.1093/bjsopen/zrac141)
Supplement: zrac141_Supplementary_Data [file zrac141_supplementary_data.docx]

# **Title**: Resectability of bilobar liver tumours after simultaneous portal and hepatic vein embolization versus portal vein embolization alone: meta-analysis

*Remon Korenblik^1,2^, *Jasper F.J.A. van Zon^1^, Bram Olij^1,2,3^, Jan Heil^4^, Maxime J.L. Dewulf^1^, Ulf. P. Neumann^1,3^, Steven. W.M. Olde Damink^1,3,5^, Christoph A. Binkert^6^, Erik Schadde^7,8^, Christiaan van der Leij^9^, Ronald. M. van Dam^1,2,3^, DRAGON trials collaborative

*First authors contributed equally

1. Department of Surgery, Maastricht University Medical Center, Maastricht, the Netherlands
2. GROW – School for Oncology and reproduction, Maastricht University, Maastricht, the Netherlands
3. Department of General, Visceral and Transplant Surgery, Uniklinik RWTH Aachen, Aachen, Germany.
4. Department of General, Visceral and Transplant Surgery, University Hospital Frankfurt, Goethe University Frankfurt, Frankfurt, Germany
5. NUTRIM - School of Nutrition and Translational Research in Metabolism, Maastricht University, Maastricht, The Netherlands
6. Department of Radiology, Cantonal Hospital Winterthur, Winterthur, Switzerland
7. Department of General, Visceral and Transplant Surgery, Klinik Hirslanden, Zurich, Switzerland
8. Department of General, Visceral and Transplant Surgery, Hirslanden Klink St. Anna Luzern, Luzern, Switzerland.
9. Department of Radiology, Maastricht University Medical Center+, Maastricht, The Netherlands.

Corresponding Author during review process and for publication:

Remon Korenblik,

Address: Universiteigssingel 50 (room 5.452) 6229 ER Maastricht,

+31637297507

remon.korenblik@mumc.nl

Corresponding Author for publication:

Ronald. M. van Dam

Maastricht UMC, Dept. of Surgery, Level 4,

PO Box 5800, 6202 AZ Maastricht, The Netherlands
+31 43 387 5492
r.vandam@mumc.nl

**Supplementary Materials - Index**

| **Supplementary Appendixes** |  |
| --- | --- |
| Appendix S1. PRISMA guidelines | *pag. 3-6* |
| Appendix S2. Search strategies | *pag. 7-9* |
| **Supplementary Figures and Tables** |  |
| Table S1 - Additional information on FLR- cut off, Definition of PHLF, Segment 4 embolization, middle hepatic vein occlusion and FLR baseline formula used in the different studies. | *pag. 10* |

**Supplementary Appendixes**

## **Appendix S1. PRISMA guidelines**

| **Topic** | **No.** | **Item** | **Location where item is reported** |
| --- | --- | --- | --- |
| **TITLE** |  |  |  |
| **Title** | 1 | Identify the report as a systematic review. | 1 |
| **ABSTRACT** |  |  |  |
| **Abstract** | 2 | See the PRISMA 2020 for Abstracts checklist | 2 |
| **INTRODUCTION** |  |  |  |
| **Rationale** | 3 | Describe the rationale for the review in the context of existing knowledge. | 3 |
| **Objectives** | 4 | Provide an explicit statement of the objective(s) or question(s) the review addresses. | 3 |
| **METHODS** |  |  |  |
| **Eligibility criteria** | 5 | Specify the inclusion and exclusion criteria for the review and how studies were grouped for the syntheses. | 4 |
| **Information sources** | 6 | Specify all databases, registers, websites, organisations, reference lists and other sources searched or consulted to identify studies. Specify the date when each source was last searched or consulted. | 4 |
| **Search strategy** | 7 | Present the full search strategies for all databases, registers and websites, including any filters and limits used. | Appendix B |
| **Selection process** | 8 | Specify the methods used to decide whether a study met the inclusion criteria of the review, including how many reviewers screened each record and each report retrieved, whether they worked independently, and if applicable, details of automation tools used in the process. | 4 |
| **Data collection process** | 9 | Specify the methods used to collect data from reports, including how many reviewers collected data from each report, whether they worked independently, any processes for obtaining or confirming data from study investigators, and if applicable, details of automation tools used in the process. | 4 |
| **Data items** | 10a | List and define all outcomes for which data were sought. Specify whether all results that were compatible with each outcome domain in each study were sought (e.g. for all measures, time points, analyses), and if not, the methods used to decide which results to collect. | 4 |
|  | 10b | List and define all other variables for which data were sought (e.g. participant and intervention characteristics, funding sources). Describe any assumptions made about any missing or unclear information. | 4 |
| **Study risk of bias assessment** | 11 | Specify the methods used to assess risk of bias in the included studies, including details of the tool(s) used, how many reviewers assessed each study and whether they worked independently, and if applicable, details of automation tools used in the process. | 4 |
| **Effect measures** | 12 | Specify for each outcome the effect measure(s) (e.g. risk ratio, mean difference) used in the synthesis or presentation of results. | 4 |
| **Synthesis methods** | 13a | Describe the processes used to decide which studies were eligible for each synthesis (e.g. tabulating the study intervention characteristics and comparing against the planned groups for each synthesis (item 5)). | - |
|  | 13b | Describe any methods required to prepare the data for presentation or synthesis, such as handling of missing summary statistics, or data conversions. | - |
|  | 13c | Describe any methods used to tabulate or visually display results of individual studies and syntheses. | - |
|  | 13d | Describe any methods used to synthesize results and provide a rationale for the choice(s). If meta-analysis was performed, describe the model(s), method(s) to identify the presence and extent of statistical heterogeneity, and software package(s) used. | - |
|  | 13e | Describe any methods used to explore possible causes of heterogeneity among study results (e.g. subgroup analysis, meta-regression). | - |
|  | 13f | Describe any sensitivity analyses conducted to assess robustness of the synthesized results. | - |
| **Reporting bias assessment** | 14 | Describe any methods used to assess risk of bias due to missing results in a synthesis (arising from reporting biases). | - |
| **Certainty assessment** | 15 | Describe any methods used to assess certainty (or confidence) in the body of evidence for an outcome. | - |
| **RESULTS** |  |  |  |
| **Study selection** | 16a | Describe the results of the search and selection process, from the number of records identified in the search to the number of studies included in the review, ideally using a flow diagram. | 5 |
|  | 16b | Cite studies that might appear to meet the inclusion criteria, but which were excluded, and explain why they were excluded. | 5 |
| **Study characteristics** | 17 | Cite each included study and present its characteristics. | 14 |
| **Risk of bias in studies** | 18 | Present assessments of risk of bias for each included study. | 15 |
| **Results of individual studies** | 19 | For all outcomes, present, for each study: (a) summary statistics for each group (where appropriate) and (b) an effect estimate and its precision (e.g. confidence/credible interval), ideally using structured tables or plots. | - |
| **Results of syntheses** | 20a | For each synthesis, briefly summarize the characteristics and risk of bias among contributing studies. | 5-6 |
|  | 20b | Present results of all statistical syntheses conducted. If meta-analysis was done, present for each the summary estimate and its precision (e.g. confidence/credible interval) and measures of statistical heterogeneity. If comparing groups, describe the direction of the effect. | 17 |
|  | 20c | Present results of all investigations of possible causes of heterogeneity among study results. | - |
|  | 20d | Present results of all sensitivity analyses conducted to assess the robustness of the synthesized results. | - |
| **Reporting biases** | 21 | Present assessments of risk of bias due to missing results (arising from reporting biases) for each synthesis assessed. | - |
| **Certainty of evidence** | 22 | Present assessments of certainty (or confidence) in the body of evidence for each outcome assessed. | - |
| **DISCUSSION** |  |  |  |
| **Discussion** | 23a | Provide a general interpretation of the results in the context of other evidence. | 7 |
|  | 23b | Discuss any limitations of the evidence included in the review. | 8 |
|  | 23c | Discuss any limitations of the review processes used. | 8 |
|  | 23d | Discuss implications of the results for practice, policy, and future research. | 9 |
| **OTHER INFORMATION** |  |  |  |
| **Registration and protocol** | 24a | Provide registration information for the review, including register name and registration number, or state that the review was not registered. | 9 |
|  | 24b | Indicate where the review protocol can be accessed, or state that a protocol was not prepared. | 9 |
|  | 24c | Describe and explain any amendments to information provided at registration or in the protocol. | - |
| **Support** | 25 | Describe sources of financial or non-financial support for the review, and the role of the funders or sponsors in the review. | 9 |
| **Competing interests** | 26 | Declare any competing interests of review authors. | 9 |
| **Availability of data, code and other materials** | 27 | Report which of the following are publicly available and where they can be found: template data collection forms; data extracted from included studies; data used for all analyses; analytic code; any other materials used in the review. | - |

*From:* Page MJ, McKenzie JE, Bossuyt PM, Boutron I, Hoffmann TC, Mulrow CD, et al. The PRISMA 2020 statement: an updated guideline for reporting systematic reviews. MetaArXiv. 2020, September 14. DOI: 10.31222/osf.io/v7gm2. For more information, visit: [www.prisma-statement.org](file:///C:\Users\jasper_zon\Documents\A-KO\Fase%202\Klinisch%20Onderzoek\SysRev%20-%20PVE%20and%20HVE\www.prisma-statement.org)

## **Appendix S2. Search strategies**

### **Search strategy in PubMed**

**P**opulation

(((((((((((((((((((((((((((((((((((((((((((((((((liver tumor) OR (liver tumour)) OR (liver neoplasm)) OR (liver metastases)) OR (liver metastasis)) OR (liver metastatic tumor)) OR (liver metastatic tumour)) OR (liver metastatic neoplasm)) OR (hepatic metastatic disease)) OR (hepatic metastatic tumor)) OR (hepatic metastatic tumour)) OR (hepatic metastatic neoplasm)) OR (hepatic metastases)) OR (hepatic metastasis)) OR (hepatic metastases colorectal)) OR (hepatic neoplasm)) OR (colorectal metastases liver)) OR (colorectal metastasis liver)) OR (colorectal liver metastases)) OR (colorectal liver metastasis)) OR (colorectal liver metastatic tumor)) OR (colorectal liver metastatic tumour)) OR (colorectal liver metastatic neoplasm)) OR (CRLM)) OR (hepatic metastases)) OR (hepatic metastasis)) OR (hepatic metastases colorectal)) OR (colorectal hepatic metastases)) OR (colorectal cancer hepatic metastases)) OR (HCC)) OR (CCC)) OR (intrahepatic cholangiocarcinoma)) OR (IHCC)) OR (perihilar cholangiocarcinoma)) OR (perihilar cholangiocarcinomas)) OR (PHCC)) OR (secondary liver tumor)) OR (secondary liver tumour)) OR (secondary liver tumors)) OR (secondary liver tumours)) OR (primary liver tumor)) OR (primary liver tumour)) OR (primary liver tumors)) OR (primary liver tumours)) OR (klatskin tumor)) OR (klatskin tumors)) OR (klatskin tumour)) OR (klatskin tumours)) OR (klatskin)) OR (((((((((((((((((((liver neoplasm[MeSH Terms]) OR (liver neoplasm, experimental[MeSH Terms])) OR (liver neoplasms[MeSH Terms])) OR (liver neoplasms, experimental[MeSH Terms])) OR (liver cancer[MeSH Terms])) OR (liver cancer, adult[MeSH Terms])) OR (liver cancers, adult[MeSH Terms])) OR (liver cancers[MeSH Terms])) OR (liver cell carcinoma, adult[MeSH Terms])) OR (liver cell carcinomas[MeSH Terms])) OR (hepatic cancer[MeSH Terms])) OR (hepatic cancers[MeSH Terms])) OR (hepatic neoplasm[MeSH Terms])) OR (hepatic neoplasms[MeSH Terms])) OR (hepatocellular carcinoma[MeSH Terms])) OR (hepatocellular carcinomas[MeSH Terms]) ) OR (klatskin tumor[MeSH Terms])) OR (cholangiocarcinoma[MeSH Terms])) OR (cholangiocarcinomas[MeSH Terms]))

**I**ntervention

(((((((((((((((((((portal and hepatic vein embolization) OR (PVE/HVE)) OR (combined portal vein and hepatic vein embolization)) OR (bi-embolization)) OR (biembolization)) OR (double vein embolization)) OR (combined simultaneous embolization of the portal vein and hepatic vein)) OR (liver venous deprivation)) OR (extended liver venous deprivation)) OR (LVD)) OR (eLVD)) OR (radiological simultaneous portohepatic vein embolization)) OR (RASPE))) OR (hepatic vein embolization)) OR (hepatic vein embolisation)) OR (portal and hepatic vein embolisation)) OR (double vein embolisation)) OR (dual embolization)) OR (dual embolisation)

**C**omparison

((((portal vein embolization) OR (PVE)) OR (portal vein ligation)) OR (PVL)) OR (portal vein embolisation)

**O**utcome

(((liver, enlarged[MeSH Terms]) OR (hypertrophy[MeSH Terms])) OR (hepatomegaly[MeSH Terms])) OR ((((((((((((((((((((((((future remnant liver volume) OR (liver hypertrophy)) OR (liver enlargement)) OR (kinetic growth rate)) OR (KGR)) OR (liver resectability)) OR (future remnant liver)) OR (FLR)) OR (liver regeneration)) OR (hypertrophy)) OR (resectability)) OR (feasibility of resection)) OR (resected)) OR (resection rate)) OR (resection feasibility)) OR (operation feasibility)) OR (feasibility of operation)) OR (operation rate)) OR (feasibility of surgery)) OR (surgery feasibility)) OR (growth rate)) OR (degree of hypertrophy)) OR (DH)) OR (hypertrophy rate))

HITS: 815

### **Search strategy in Web of Science**

**P**opulation

(((((((((((ALL=(liver tumor)) OR ALL=(liver tumour)) OR ALL=(liver neoplasm)) OR ALL=(liver metastases)) OR ALL=(liver metastasis)) OR ALL=(colorectal liver metastases)) OR ALL=(colorectal liver metastasis)) OR ALL=(liver cancer$)) OR ALL=(liver cell carcinoma)) OR ALL=(liver cell carcinomas)) OR ALL=(hepatocellular carcinoma)) OR ALL=(Klatskin tumor)

**I**ntervention

(((((((((((ALL=(portal and hepatic vein embolization)) OR ALL=(Combined portal vein and hepatic vein embolization)) OR ALL=(bi-embolization)) OR ALL=(biembolization)) OR ALL=(double vein embolization)) OR ALL=(Combined simultaneous embolization of the portal vein and hepatic vein)) OR ALL=(liver venous deprivation)) OR ALL=(LVD)) OR ALL=(extended liver venous deprivation)) OR ALL=(eLVD)) OR ALL=(Radiological Simultaneous Portohepatic Vein Embolization)) OR ALL=(RASPE)

**C**omparison

(((ALL=(portal vein embolization)) OR ALL=(pve)) OR ALL=(portal vein ligation)) OR ALL=(PVL)

**O**utcome

(((((((((ALL=(future remnant liver)) OR ALL=(FLR)) OR ALL=(liver hypertrophy)) OR ALL=(liver enlargement)) OR ALL=(liver regeneration)) OR ALL=(kinetic growth rate)) OR ALL=(KGR)) OR ALL=(liver resection)) OR ALL=(liver hypertrophy)) OR ALL=(liver regeneration)

HITS: 1354

In Web of Science, "MeSH terms" have not been used, because that option is (not known to us) available.

### **Search strategy in EMBASE**

**P**opulation

liver cell carcinoma/ or liver cancer/ or colorectal liver metastasis/ or liver metastasis/ or liver tumor/ or Klatskin tumor/ or (liver tumour or hepatic tumor or hepatic tumour or liver neoplasm or hepatic neoplasm or hepatocellular carcinoma or colorectal liver metastatic tumor or colorectal liver metastatic tumour or colorectal liver metastatic neoplasm or CRLM or hepatic metastases or hepatic metastases colorectal or colorectal cancer hepatic metastases or HCC or CCC or intrahepatic cholangiocarcinoma or intrahepatic cholangiocarcinomas or IHCC or perihilar cholangiocarcinoma or perihilar cholangiocarcinomas or PHCC or secondary liver tumors or primary liver tumors or klatskin tumor or klatskin tumors or klatskin).af.

**I**ntervention

Artificial embolization/ or ((Portal and hepatic vein embolization) or (Combined portal vein and hepatic vein embolization) or "PVE HVE" or Liver venous deprivation or LVD or extended venous liver deprivation or eLVD or radiological simultaneous portohepatic vein embolization or RASPE or dual embolization or biembolization or bi-embolization or double vein embolization).af.

**C**omparison

(portal vein embolization or portal vein occlusion or PVE or portal vein ligation or PVL).af.

**O**utcome

liver resection/ or liver hypertrophy/ or liver regeneration/ or hepatomegaly/ or (kinetic growth rate or KGR or future remnant liver or FLR or future remnant liver volume or liver enlargement or resectability or feasibility of resection of resected or resection rate or resection feasibility or operation feasibility or feasibility of operation or operation rate or feasibility of surgery or surgery feasibility or growth or degree of hypertrophy or DH or hypertrophy rate).af.

Hits: 456

Because of the low number of hits (38) in the initial search, specific EMTREE terms were added to the search - based on expertise that certain articles should be included - so that 424 articles were eventually retrieved. By applying this method we believe that we included more relevant articles from EMBASE.

**Supplementary Figures and Tables**

**Table S1. Additional information on FLR- cut off, Definition of PHLF, Segment 4 embolization, middle hepatic vein occlusion and FLR baseline formula used in the different studies.**

| **Author** | **Selection FLR% criteria*** | **Definition of PHLF** | **S4 embolization** | **MHV occlusion** | **FLR baseline formula** |
| --- | --- | --- | --- | --- | --- |
| **Hocquelet et al. (2018)** | FLR <40% | n.r. | not performed | not performed | %FLR = FLR/TLV^##^ |
| **Panaro et al. (2019)** | FLR <25-30% (normal liver); FLR <35-40% (underlying liver disease) | ISGLS** | n.r. | n.r. | n.r. |
| **Le Roy et al. (2020)** | FLR <25% (normal liver); FLR <40% (underlying liver disease) | ISGLS | n.r. | 3 patients | %FLR = FLR/TLV^#^ |
| **Kobayashi et al. (2020)** | FLR <30% (normal liver); FLR <35% (underlying liver disease) | n.r. | 3 patients | 2 patients | %sFLR = FLR/sTLV^##^ |
| **Laurent et al. (2020)** | PVE: FLR <30%  PVE/HVE: FLR <25% | ISGLS | not performed | 8 patients (21.6%) | %FLR = FLR/TLV^#^ |
| **Guiu et al. (2020)** | FLR <30% or 99mTc-mebrofenin clearance rat <2.69%/min/m2 | “50-50” criteria*** | not performed | n.r. | %FLR = FLR/TLV^#^ |
| **Heil et al. (2021)** | FLR <30% | ISGLS | not performed | 14 patients | %sFLR = FLR/sTLV^###^ |
| **Boning et al. (2022)** | n.r. | n.r. | n.r. | Not performed | n.r. |
| **Guiu et al. (2016)** | FLR <25% (normal liver); FLR <40% (underlying liver disease) | n.r. | not performed | n.r. | %FLR = FLR/TLV^#^ |
| **Guiu et al. (2017)** | FLR <25% (normal liver) or 99mTc-mebrofenin clearance rat <2.69%/min/m2 | “50-50” criteria | n.r. | n.r. | %sFLR = FLR/sTLV^##^ |
| **Le Roy et al. (2017)** | FLR <25% (normal liver); FLR <40% (underlying liver disease) | “50-50” criteria, Mullen criteria, or ISGLS | not performed | 3 patients | FLR/TLV^##^ |
| **Chebaro et al. (2021)** | n.r. | n.r. | n.r. | n.r. | n.r. |
| **Ghosn et al. (2021)** | FLR <35-40% | n.r. | n.r. | not performed | %FLR = FLR/TLV^##^ |
| **Cassese et al. (2022)** | FLR <25% (normal liver); FLR <30% (steatosis/chemotherapy demaged livers); FLR <40% (cirrhosis and cholestasis) | ISGLS | n.r. | Some patients, depending on the baseline FLR and the type of surgery (resection of  segment IV) | LR-V share = FRL-V/(eTLV- TV) x 100## |

*PVE/HVE: portal and hepatic vein embolization; PHLF: post-hepatectomy liver failure; PVE: portal vein embolization; MHV: middle hepatic vein; n.r.: not reported; FLR: future liver remnant; sFLR: standardized future liver remnant; TLV: total liver volume; sTLV: standardized total liver volume*

** Baseline %FLR criteria were not specifically specified for either PVE or PVE/HVE, otherwise this was mentioned.*

*** Definition of PHLF presented by the International Study Group of Liver Surgery [x]*

**** ”50-50” criteria was presented by Balzan et al. (2005)*

*# Formula for TLV was not mentioned*

*## sTLV = -794.41 + 1267.28 * body surface area*

*### TLV = 18.51 * bodyweight (kg) + 191.8*
